# Machine learning models for predicting the onset of chronic kidney disease after surgery in patients with renal cell carcinoma

**DOI:** 10.1186/s12911-024-02473-8

**Published:** 2024-03-22

**Authors:** Seol Whan Oh, Seok-Soo Byun, Jung Kwon Kim, Chang Wook Jeong, Cheol Kwak, Eu Chang Hwang, Seok Ho Kang, Jinsoo Chung, Yong-June Kim, Yun-Sok Ha, Sung-Hoo Hong

**Affiliations:** 1 Department of Medical Informatics, College of Medicine, The Catholic University of Korea, 06591 Seoul, Korea; 2 Department of Biomedicine & Health Sciences, The Catholic University of Korea, 06591 Seoul, Korea; 3 Department of Urology, Seoul National University College of Medicine, Seoul National University Bundang Hospital, 13620 Seongnam, Korea; 4 Department of Urology, Seoul National University College of Medicine, Seoul National University Hospital, 03080 Seoul, Korea; 5 Department of Urology, Chonnam National University Medical School, 61469 Gwangju, Korea; 6 Department of Urology, Korea University School of Medicine, 02841 Seoul, Korea; 7 Department of Urology, National Cancer Center, 10408 Goyang, Korea; 8 Department of Urology, Chungbuk National University College of Medicine, 28644 Cheongju, Korea; 9 Department of Urology, College of Medicine, Chungbuk National University, 28644 Cheongju, Korea; 10 Department of Urology, School of Medicine, Kyungpook National University Chilgok Hospital, Kyungpook National University, 41404 Daegu, Korea; 11 Department of Urology, Seoul St. Mary’s Hospital, College of Medicine, The Catholic University of Korea, Seoul, Republic of Korea

**Keywords:** Renal cell carcinoma, Machine learning, Chronic kidney disease, KOrean Renal Cell Carcinoma, Gradient boost

## Abstract

**Background:**

Patients with renal cell carcinoma (RCC) have an elevated risk of chronic kidney disease (CKD) following nephrectomy. Therefore, continuous monitoring and subsequent interventions are necessary. It is recommended to evaluate renal function postoperatively. Therefore, a tool to predict CKD onset is essential for postoperative follow-up and management.

**Methods:**

We constructed a cohort using data from eight tertiary hospitals from the Korean Renal Cell Carcinoma (KORCC) database. A dataset of 4389 patients with RCC was constructed for analysis from the collected data. Nine machine learning (ML) models were used to classify the occurrence and nonoccurrence of CKD after surgery. The final model was selected based on the area under the receiver operating characteristic (AUROC), and the importance of the variables constituting the model was confirmed using the shapley additive explanation (SHAP) value and Kaplan-Meier survival analyses.

**Results:**

The gradient boost algorithm was the most effective among the various ML models tested. The gradient boost model demonstrated superior performance with an AUROC of 0.826. The SHAP value confirmed that preoperative eGFR, albumin level, and tumor size had a significant impact on the occurrence of CKD after surgery.

**Conclusions:**

We developed a model to predict CKD onset after surgery in patients with RCC. This predictive model is a quantitative approach to evaluate post-surgical CKD risk in patients with RCC, facilitating improved prognosis through personalized postoperative care.

## Introduction

Detection of kidney cancer has improved because of the proliferation of imaging tests such as ultrasound and computed tomography. The kidney is ninth among the most common primary cancer sites, with renal cell carcinomas (RCCs) constituting approximately 90% of these cases [1]. Metastatic diseases affect approximately 20–30% of those diagnosed with RCC, which eventually claims the lives of over 40% of these patients [1–3].

Most patients with localized RCC undergo radical or partial nephrectomy [4–6]. Although radical nephrectomy offers excellent oncological outcomes for confined malignancies, it is associated with complications [7]. The literature features prominent concerns about the risk of developing chronic kidney disease (CKD) postoperatively following a reduction in nephron mass [8]. Individuals diagnosed with CKD, which is characterized by an estimated glomerular filtration rate (eGFR) of < 60 ml/min/1.73 m^2^, frequently experience progression to kidney failure, complications due to diminished renal function, or cardiovascular disease and, in some cases, death [9, 10].

Current guidelines from the European Association of Urology and American Urological Association recommend assessing renal function in patients who have undergone surgery for RCC [11]. Renal function in some patients progressively declines following nephrectomy; 1–2% of patients who undergo this procedure ultimately require renal replacement therapy as a result of developing end-stage kidney disease (ESKD) [12, 13]. Hence, it is crucial to identify patients at an elevated risk of developing CKD following nephrectomy.

Research on leveraging machine learning (ML) has emerged with advances in computer technology. Currently, research on medical data for cancer prediction using ML is being intensively pursued [14–17]. Research on ML in patients with CKD is intensifying. As of 2021, 28 studies have focused on utilizing ML for disease prognosis analysis, and 21 studies have been dedicated to disease diagnostic analysis [18]. However, there is a notable lack of studies predicting CKD in patients who have undergone RCC surgery. Most existing studies target the general patient population or focus on utilizing ML for prognosis rather than CKD prediction [19–21]. Risk and odds ratios have been assessed in studies on CKD onset in RCC surgery patients using regression and survival analyses; however, they cannot provide direct probability calculations [4, 22–25]. If ML is employed, which calculates probabilities and identifies influential factors, it can serve as a diagnostic tool.

In this study, we assessed whether the risk factors identified in prior research are consistent in Korean patients with RCC. Further, we introduced an ML model that forecasts the likelihood of CKD based on a combination of these factors. As our algorithm was formulated using a multicenter dataset sourced from a top-tier Korean hospital, it represents the features of Korean RCC patients without any distortion. Moreover, to our knowledge, our study pioneered the use of ML to predict CKD in patients with RCC. By employing the algorithm we crafted, detecting CKD in patients at an early postoperative stage becomes feasible, allowing for timely and appropriate treatment, enhancing their overall outcome.

## Materials and methods

### Study population

A web-based database system called the Korean Renal Cell Carcinoma (KORCC) was created to gather basic demographic and clinicopathological data on a sizable cohort of RCC patients in Korea. It was retrospectively built using data from eight hospitals from 1990 to the present [26]. This database was approved by the Ethics Committee of Seoul National University Bundang Hospital (IRB No.: B1202/145 − 102). Using this database, we obtained data from 9598 patients with RCC, encompassing 214 variables. These variables comprised basic demographic factors such as age, sex, height, and weight and clinicopathological features, including clinical stage, pre- and post-surgical test results, and pathological stage. Furthermore, the outcomes were established based on assessing postoperative eGFR levels. CKD was defined as a postoperative eGFR value dropping below 60 ml/min/1.73 m^2^ [23], which we designated as the outcome. Notably, the eGFR value referred to here was computed using the CKD-EPI equation. The study protocol was approved by the Institutional Review Board (IRB) of the Catholic University of Korea (IRB No. KC23ZIDI0683). The IRB of the Catholic University of Korea waived the requirement for informed consent because this study was retrospective, and personal information in the data were blinded.

### Variable selection

The variable selection process was meticulously structured into three sequential phases. Initially, we focused on existing literature addressing postoperative CKD in patients with RCC, leading us to undertake a comprehensive meta-analysis [4, 22–25]. This study identified 38 notable variables from the previous studies. From this pool, 17 were found to be compatible with our dataset. In the second phase, rigorous statistical evaluations were performed to verify the authenticity of each variable and monitor any missing data points. A *p*-value threshold of 0.05 was our yardstick for validation. To differentiate between the non-post-CKD and post-CKD cohorts, continuous variables underwent t-test evaluations, whereas categorical counterparts were scrutinized using chi-squared tests, ensuring that we highlighted statistically significant variances. In the last phase, with valuable input from urology specialists, we integrated an additional variable, “smoking,” culminating in a final tally of 12 pivotal variables for our analysis. The final variables included sex, smoking status, history of diabetes mellitus (DM), presence of hypertension (HTN), European Cooperative Oncology Group (ECOG) performance score, hemoglobin (Hb) level, creatinine level, albumin level, calcium level, preoperative eGFR value, tumor size, and tumor location.

Gender was categorized into two groups: male and female, and utilized as a variable in all associated research. Diabetes and hypertension were classified based on their presence or absence. Smoking status was divided into three categories: non-smokers, current smokers, and former smokers who do not currently smoke. The ECOG status, with scores ranging from 0 to 4, indicated physical activity ability; a higher score signifies greater functioning. The ‘Tumor Location’ variable reflects the extent of the tumor’s occupancy on the kidney’s surface. Variables such as Hb, Creatinine, Albumin, Calcium, eGFR, and Tumor Size were continuous, derived from test measurements. All variables, with the exception of Hb, Tumor Size, and Tumor Location, were identified as significant factors in prior research [4, 22–25].

### Data screening

**Fig. 1:**
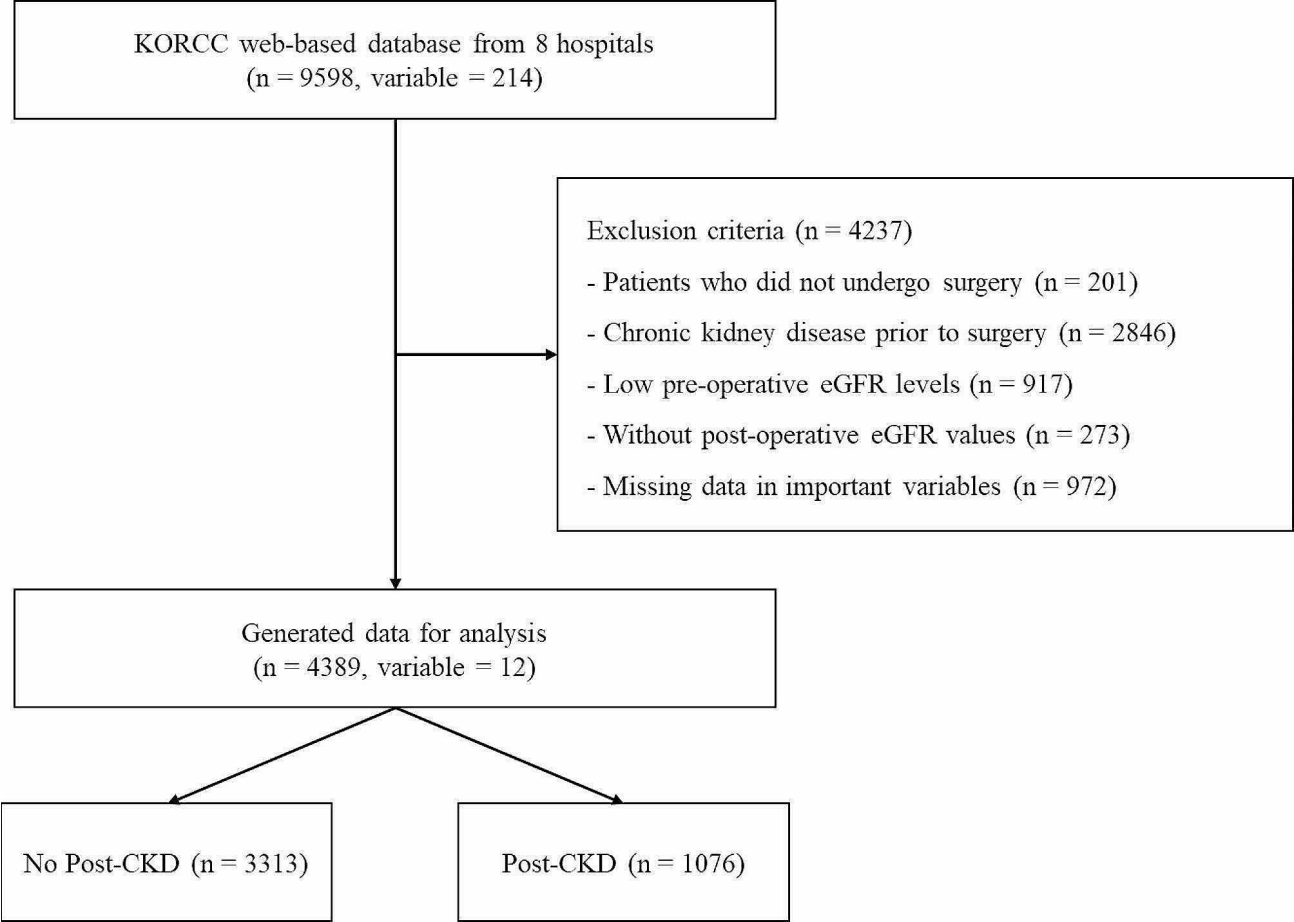
Data flowchart for analysis

Of the 9,598 patients with RCC, 201 who did not undergo surgery were excluded. Subsequently, we excluded 2,846 patients diagnosed with CKD before surgery, 917 patients with preoperative eGFR levels below 60, 273 patients lacking postoperative eGFR readings, and 972 patients with missing data. Of the remaining 4389 patients, 1,076 developed CKD post-surgery, while 3313 did not exhibit postoperative CKD (Fig. 1).

### Data splitting and SMOTE for imbalanced datasets

We divided the data at a 7:3 ratio to train and evaluate our model to create two distinct datasets. Given the relatively small number of patients in the post-CKD group, there was a potential for class imbalance. In other words, if trained as is, the model might heavily lean toward the larger no-post-CKD group, ultimately forming a model that predominantly predicts the no-post-CKD group. Techniques such as oversampling, undersampling, and SMOTE can be employed [27–29]. We opted for the SMOTE method, known for its widespread use and efficacy, to equalize the training data to a patient group ratio of 1:1 (Table 1).

**Table 1:** Dataset distribution before to and following the SMOTE application

	Training set (*n* = 3072)		Test set (*n* = 1317)	
	no-post-CKD group, *n* (%)	post-CKD group, *n* (%)	no-post-CKD group, *n* (%)	post-CKD group, *n* (%)
Before	2319 (75.49)	753 (24.51)	994 (75.47)	323 (24.53)
After	2319 (50.00)	2319 (50.00)	994 (75.47)	323 (24.53)

### Model development and validation

In this study, we evaluated the efficacy of several prominent ML classifiers: kernel support vector machine (SVM) [30], logistic regression [31], decision tree [32], k-nearest neighbor (KNN) [33], random forest [34], gradient boost [35], AdaBoost [34, 36], XGBoost [37], and LightGBM [38]. For each classifier, we derived four metrics: sensitivity, specificity, accuracy, F1-score, and area under the receiver operating characteristic (AUROC) [39]. The model delivering the superior performance was chosen based on its AUROC score, which is a crucial metric for assessing classifier effectiveness. All statistical analyses and algorithmic developments were performed using Python (version 3.9.7).

Using the final selected model, we examined the primary factors using the SHapley Additive exPlanations (SHAP) values. SHAP values serve as a representative element of explainable AI, offering a method to gauge the contribution of each variable within the model [40]. Thus, we could discern which variables played a significant role in the model and whether their impact on the outcome was positive or negative. This study aimed to determine the influence of each variable.

## Results

### Patient characteristics

We assessed the differences in patient attributes and the distribution of individual variables between the post-CKD and non-post-CKD cohorts (Table 2). A higher proportion of patients who developed CKD after surgery were male (73.4% vs. 68.2% in the non-CKD group). The percentage of patients with DM was 20.7% in the postoperative CKD group and 12.9% in the no-post-CKD group. Similarly, the incidences of hypertension (HTN) were 47.9% and 34.4% in the post-CKD and non-post-CKD groups, respectively. These results are in line with those of previous research [4, 22, 24, 25]. The average eGFR values (ml/min/1.73 m^2^) in the no-post-CKD and post-CKD groups after surgery were 94.7 and 77.7, respectively. Additionally, the average tumor size(cm) before surgery was 38.6% in the no post-CKD group and 52.4 for the post-CKD group. This suggests that patients in the post-CKD group had lower eGFR and larger tumor size before surgery. The distributions of the other variables are listed in Table 2.

**Table 2:** Baseline characteristics of the patients (*N* = 4389)

Variable	no-post-CKD group (*n* = 3313)	post-CKD group (*n* = 1076)	*p*-value
**Sex**			0.001
Male	2260 (68.2%)	790 (73.4%)	
Female	1053 (31.8%)	286 (26.6%)	
**DM**			< 0.001
None	2885 (87.1%)	853 (79.3%)	
DM	428 (12.9%)	223 (20.7%)	
**HTN**			< 0.001
None	2172 (65.6%)	561 (52.1%)	
HTN	1141 (34.4%)	515 (47.9%)	
**Smoking**			0.194
Non-smoker	1877 (56.7%)	597 (55.5%)	
Ex-smoker	845 (25.5%)	261 (24.3%)	
Current smoker	591 (17.8%)	218 (20.3%)	
**ECOG**			< 0.001
0	2794 (84.3%)	829 (77.0%)	
1	481 (14.5%)	221 (20.5%)	
2	31 (0.9%)	20 (1.9%)	
3	3 (0.1%)	5 (0.5%)	
4	4 (0.1%)	1 (0.1%)	
**Hb** (g/dL)	13.8 ± 1.7	13.7 ± 1.7	0.017
**Creatinine** (mg/dL)	0.8 ± 0.2	1.0 ± 0.2	< 0.001
**Albumin** (g/dL)	4.4 ± 0.4	4.3 ± 0.4	< 0.001
**Calcium** (mg/dL)	9.3 ± 0.9	9.2 ± 0.6	< 0.001
**eGFR** (ml/min/1.73${\text{m}}^{2}$)	94.7 ± 15.6	77.7 ± 12.5	< 0.001
**Tumor size** (cm)	38.6 ± 26.8	52.4 ± 27.9	< 0.001
**Tumor location**			< 0.001
exophytic	1694 (51.1%)	564 (52.4%)	
mesophytic	560 (16.9%)	133 (12.4%)	
endophytic	676 (20.4%)	167 (15.5%)	
hilar	337 (10.2%)	151 (14.0%)	
unknown	46 (1.4%)	61 (5.7%)	

### Model performance

We applied 12 variables to nine ML models and calculated their accuracy, specificity, sensitivity, AUROC, and F1 scores to compare their performances. In this study, the final model was selected based on the AUROC value and F1 score, considering data imbalance, sensitivity, and specificity. A grid search was conducted for each ML model to determine the optimal hyperparameters. Table 3 lists each model’s intended hyperparameters. Using the best-selected hyperparameters, we assessed the degree to which each model performed; the results are listed in Table 4.

**Table 3:** Hyperparameter optimization using grid search

Algorithms	Hyperparameters
Kernel SVM	kernel: (linear, rbf*) C: (0.1, 1, 10*) gamma: (0.1*, 0.5, 1)
Logistic regression	C: (0.1, 1, 10, 100*, 1000)
Decision tree	max_depth: (1, 5, 10, 15*, 20) min_samples_split: (1, 5, 10*, 15, 20)
KNN	n_neighbors: (1*, 2, 3, 4, 5)
Random forest	n_estimators: (10, 100, 1000, 10,000*) max_depth: (1, 5, 10, 15, 20*)
Gradient boost	n_estimators: (10, 100, 500, 1000*, 5000) learning_rate: (0.01, 0.05*, 0.1, 0.5)
AdaBoost	n_estimators: (10, 100, 500, 1000*, 5000) learning_rate: (0.01, 0.05, 0.1, 0.5*)
XGBoost	n_estimators: (10, 100*, 500, 1000, 5000) learning_rate: (0.01, 0.05, 0.1, 0.5*)
LightGBM	n_estimators: (10, 100, 500*, 1000, 5000) learning_rate: (0.01, 0.05*, 0.1, 0.5)

* Optimal parameters obtained through a grid search

**Fig. 2:**
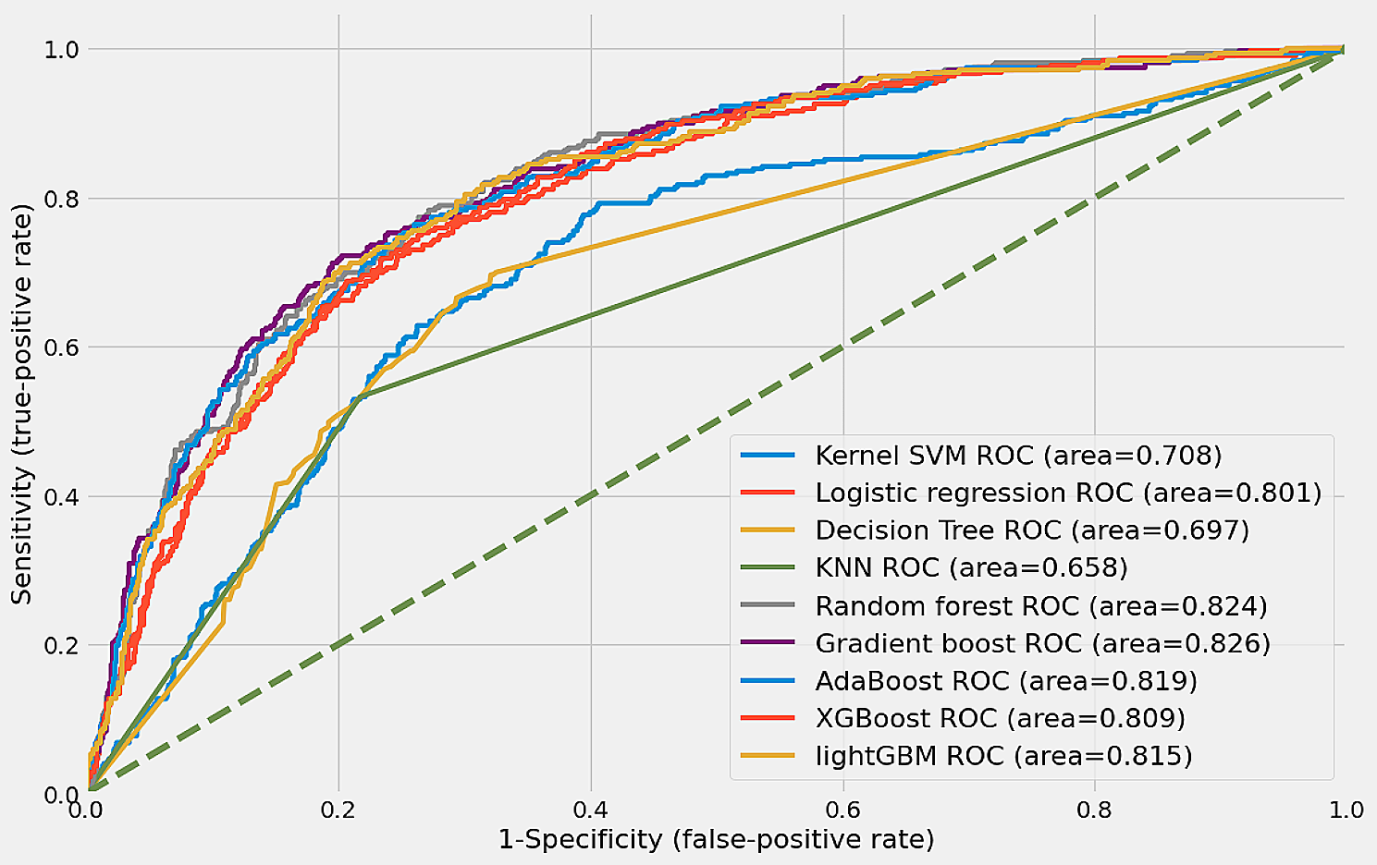
Receiver operating characteristic (ROC) curves of the ML models

We assessed the performance of each ML model using the optimized parameters, and the results comparing the sensitivity, specificity, accuracy, AUROC, and F1 scores are presented in Table 4. Figure 2 shows the receiver operating characteristic (ROC) curves for the nine ML models. A larger area under the curve indicates a higher AUROC value, indicating a superior performance. Based on the AUROC, the Gradient boost was the top-performing model. Gradient boost demonstrated a predictive performance with a sensitivity of 0.594, specificity of 0.877, accuracy of 0.808, AUROC of 0.826, and F1 score of 0.603.

**Table 4:** Performance of the ML models

Model	Sensitivity	Specificity	Accuracy	AUROC	F1-score
Kernel SVM	0.424	0.824	0.726	0.708	0.431
Logistic regression	0.700	0.766	0.749	0.801	0.578
Decision tree	0.520	0.789	0.723	0.697	0.479
KNN	0.533	0.783	0.721	0.658	0.484
Random forest	0.650	0.830	0.786	0.824	0.598
Gradient boost	0.594	0.877	0.808	0.826	0.603
AdaBoost	0.560	0.878	0.800	0.818	0.579
XGBoost	0.529	0.863	0.781	0.809	0.543
LightGBM	0.536	0.863	0.783	0.815	0.547

To ascertain the significance of the performance of the trained models, post-hoc McNemar’s Chi-Squared testing was conducted [41]. The models were compared against the Gradient boost model, which exhibited the highest AUROC. This comparison was based on the prediction results for the test set. It was observed that the Kernel SVM (*p* < 0.001), Logistic Regression (*p* < 0.001), Decision Tree (*p* < 0.001), and KNN (*p* < 0.001) all showed statistically significant differences when compared to the Gradient boost model. However, no significant differences were found with the Random forest (*p* = 0.921), AdaBoost (*p* = 0.555), XGBoost (*p* = 0.935), and LightGBM (*p* = 0.793) models. These models, all belonging to the same family of tree-based ensemble methods, presented challenges in differentiation, leading us to verify the outcomes through the analysis of confusion matrices [42].

**Fig. 3:**
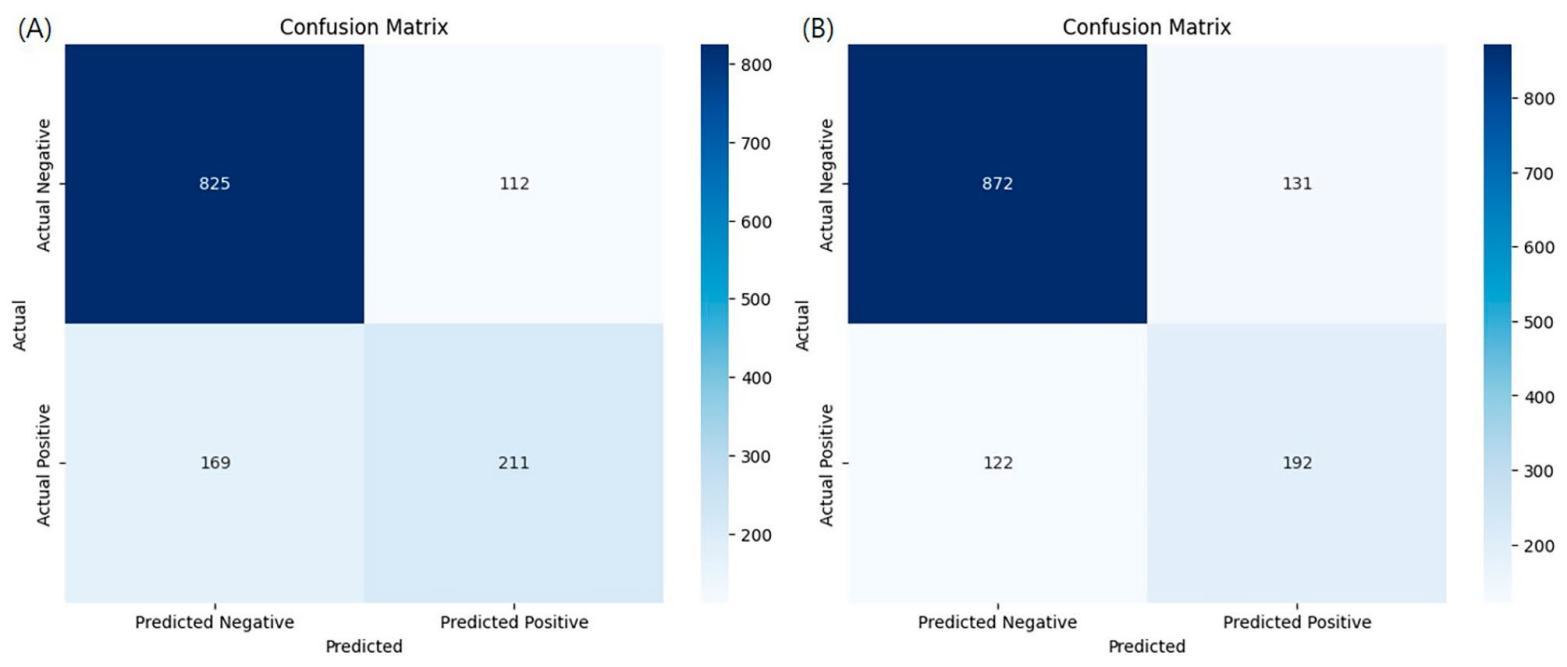
Confusion matrix of the top models: **a** Random forest; **b** Gradient boost

As depicted in Fig. 2, the performance of the Gradient boost, which showed the highest efficacy, was compared with that of the Random forest, the model with the second-highest performance, using their respective confusion matrices (Fig. 3). In terms of True Negatives (TN), Gradient boost identifies approximately 47 more instances correctly compared to Random forest. However, it is observed that Gradient boost predicts 19 fewer True Positives (TP) than Random forest. Given that the primary objective is to accurately identify diseases within imbalanced clinical data, models with higher AUROC and recall values can be considered to perform better [43]. The recall rates for each model are 55.5% for Random forest and 61.2% for Gradient boost, respectively. Consequently, it can be concluded that Gradient boost demonstrated superior predictive accuracy over Random forest.

### Visualization of feature importance

**Fig. 4:**
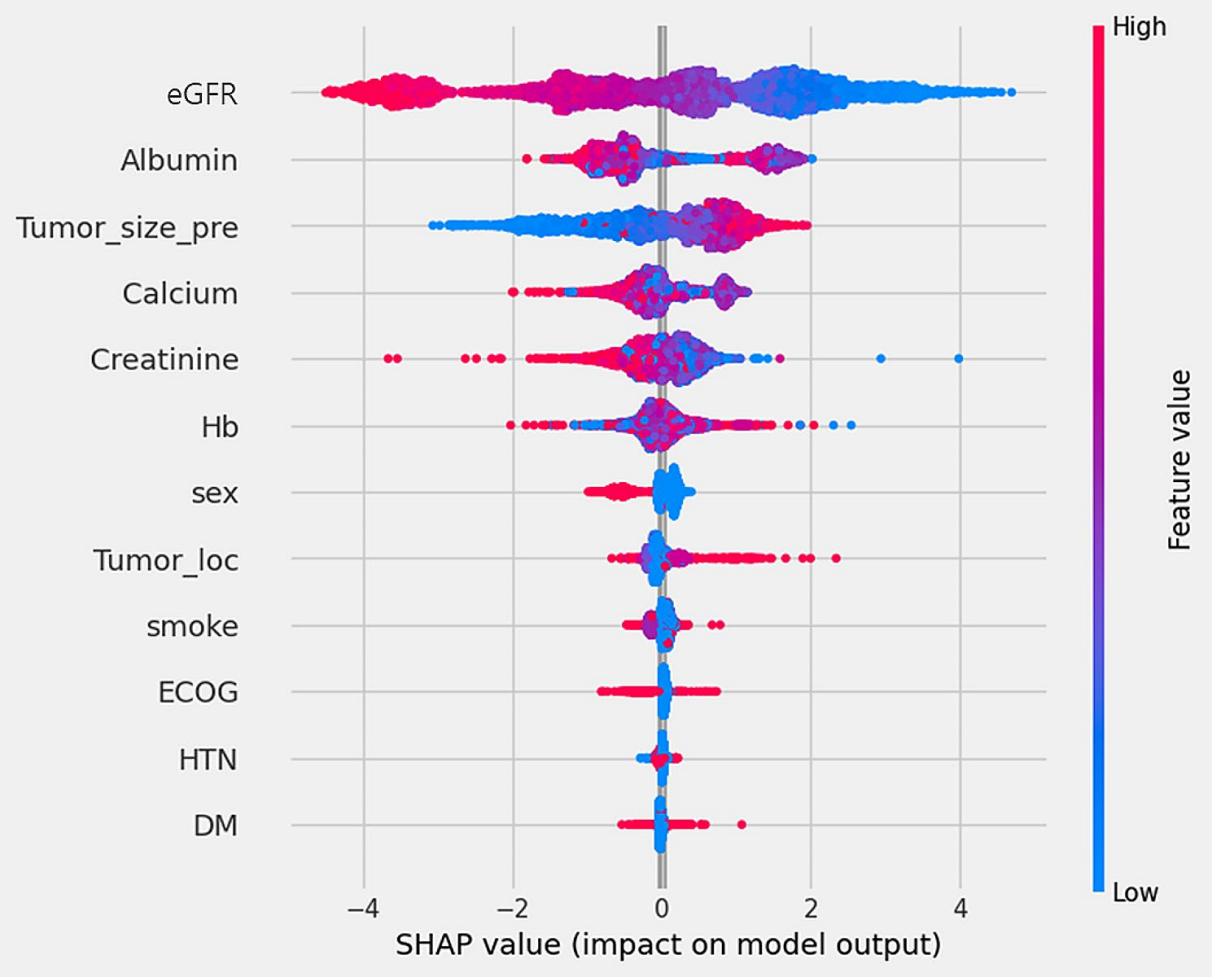
SHAP value of the Gradient boost model

SHAP was used to visually explain the variables constituting the model and verify their impact on CKD after surgery in patients with RCC. Figure 4 shows the SHAP values. The y-axis shows the importance of the model, with the most important variables at the top. The x-axis represents the exponent that responds to the effects of each variable. Here, the red points indicate high-risk values, and the blue points indicate low-risk values. Thus, it was related to the prediction that the occurrence of CKD after surgery would be higher when the eGFR value was lower, albumin was lower, tumor size was larger, calcium level was lower, creatinine level was lower, and Hb level was higher.

**Fig. 5:**
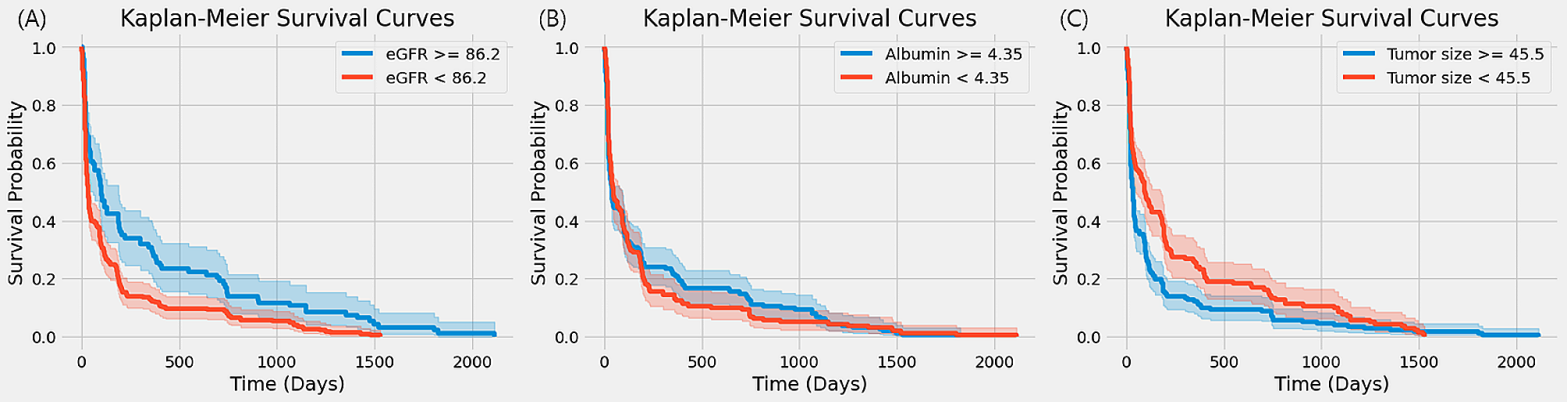
Kaplan–Meier curves for the top variables: **a** eGFR; **b** Albumin; **c** Tumor size

We conducted Kaplan-Meier (K-M) survival analyses for the most influential variables: eGFR, Albumin, and Tumor Size [25]. To facilitate a clear comparison between managed and unmanaged groups, we established criteria based on demographic data. Specifically, eGFR was divided into higher and lower groups using a threshold of 86.2, Albumin using 4.35, and Tumor Size using 45.5. In Fig. 5, K-M survival curves were then drawn to ascertain the actual impact of these variables on CKD development. While the survival rate generally dropped below 20% within a year post RCC surgery, it was observed that groups with an eGFR value higher than 86.2 maintained a survival rate above 20% for nearly two years.

## Discussion

To our knowledge, this is the first study to predict postoperative CKD in patients with RCC using an ML model, underlining its significance. Nephrectomy and CKD share a pronounced correlation [22]. Consequently, preliminary research has been conducted to ascertain these relationships and associated risk factors using statistical methods [4, 8, 22–25]. However, no prior studies have harnessed ML techniques to predict postoperative CKD, primarily because of the challenge of amassing adequate data from a single institution for ML applications. In our endeavor, we analyzed data from 4389 patients with RCC gathered from eight top-tier hospitals in our country using the KORCC database. We successfully created an algorithm that uses 12 factors for predicting the probability of postoperative CKD in patients with RCC. Gradient boost demonstrated the best performance among the nine models employed, with an AUROC of 0.826 and an f1 score of 0.603.

Nevertheless, a salient limitation of such ML models is their intricate interpretability [44]. To address this, we utilized the SHAP value to discern feature importance. Our results indicated that preoperative eGFR was the most important variable, followed by albumin level, tumor size, and calcium level (Fig. 4). According to prior studies, preoperative eGFR levels were identified as a significant factor in predicting postoperative CKD risk among patients [22–24]. Our data distribution showed a distinct difference in the average preoperative eGFR levels between the no-post-CKD group at 94.7 and the post-CKD group at 77.7. The results of the SHAP values also underscore their prominence, showing the highest significance among the variables. Additionally, albumin and creatinine, which emerged as the second and fifth most influential variables, respectively, have been underscored in prior studies as contributing factors to the onset of postoperative CKD [22]. In our dataset, we observed that the average albumin level was notably lower in the post-CKD group than in the no-post-CKD group. Conversely, the average creatinine levels were slightly elevated in the post-CKD group compared to the no-post-CKD group. The influence of preoperative tumor size on the occurrence of postoperative CKD was verified using SHAP values in our study. Previous research has also indicated that preoperative tumor sizes are notably larger in the post-CKD group than in the no-post-CKD group [22]. In line with these findings, our data showcased that the average tumor sizes in the no-post-CKD and post-CKD groups were 38.6 and 52.4 cm, respectively, highlighting a discernible difference. Our study highlights the significant influence of calcium levels. While previous research has indicated calcium as a significant variable related to preoperative CKD occurrence, it did not highlight the same correlation with postoperative CKD onset [22]. Nonetheless, in our study, slightly higher calcium levels were observed in the no-post-CKD group than in the control group.

In this analysis, a preliminary examination of albumin and calcium levels revealed no apparent significant difference between the post-CKD group and the no-post-CKD group, with an average difference of merely 0.1. However, a deeper investigation using the t-test statistical method uncovered significant disparities. The influence of sample size on statistical significance becomes evident here [45, 46]. Despite the nominal difference in mean albumin levels—merely 0.1—between the two groups, the t-test yielded a t-value exceeding 4, indicative of a significantly low *p*-value. This outcome illustrates that the significance of variables cannot be adequately assessed by the difference in sample means alone. In a similar vein, calcium levels, despite also presenting a mean difference of 0.1, were found to be significant upon t-test analysis. Furthermore, the machine learning-based SHAP value analysis also identified albumin as a highly influential factor. This underscores the importance of utilizing both statistical and machine learning approaches to fully understand the subtleties in data, especially when initial observations might suggest otherwise [47].

Furthermore, to gain an intuitive understanding of the impact on CKD development, Kaplan-Meier survival analyses were performed on the identified variables [25]. This analysis particularly focused on eGFR, Albumin, and Tumor Size, which emerged as the most influential factors. It was observed that the presence or absence of management for these factors significantly affects the incidence of CKD following RCC surgery.

Our dataset mirrored the distribution characteristics of the risk factors identified in most previous studies. Another strength of our research is the utilization of data from eight different institutions, which helps mitigate potential bias. Additionally, missing values were not permitted for the chosen variables. Employing missing-value imputation techniques for the 972 excluded patients may pave the way for developing a more robust model [48, 49].

## Conclusions

We developed a predictive model using ML algorithms to predict the onset of CKD in patients after partial or radical nephrectomy. Gradient boost exhibited the highest performance among the ML models, with an AUROC of 0.826. Using this predictive model, we calculated the likelihood of postoperative CKD occurrence in each patient with RCC. Moreover, this model can improve the prognosis of CKD in patients through tailored postoperative care and appropriate treatment.

## Data Availability

The datasets used and analyzed during the current study are available from the corresponding author on reasonable request.
